# High glucose mediates NLRP3 inflammasome activation via upregulation of ELF3 expression

**DOI:** 10.1038/s41419-020-2598-6

**Published:** 2020-05-21

**Authors:** Jing Wang, Xuefang Shen, Jing Liu, Wankun Chen, Fengfeng Wu, Weifei Wu, Zhipeng Meng, Minmin Zhu, Changhong Miao

**Affiliations:** 1 Department of Anesthesiology, Fudan University Shanghai Cancer Center, Department of Oncology, Shanghai Medical College, Fudan University, shanghai, 200032 China; 2 Department of Anaesthesiology, Huzhou Central Hospital, Affiliated Central Hospital HuZhou University, 198 Hongqi Road, Huzhou, Zhejiang People’s Republic of China; 3 Department of Anaesthesiology, Huzhou Maternal and Child Health Care Hospital, Huzhou, 313000 Zhejiang China; 4 Department of Anesthesia, Zhongshan Hospital, Fudan University, Shanghai, 200032 China; 5 Department of Orthopaedics, Huzhou Central Hospital, Huzhou, 313000 Zhejiang China; 6 Department of Nephrology, Huzhou Central Hospital, Huzhou, 313000 Zhejiang China

**Keywords:** Peripheral vascular disease, Diabetes complications

## Abstract

Microtubule affinity regulating kinase 4 (MARK4) plays a crucial role in the regulation of NOD-like receptor pyrin domain 3 (NLRP3) inflammasome activation, which leads to the generation of bioactive interleukin (IL)-1β and IL-18. E74-like ETS transcription factor 3 (ELF3) participates in endothelial inflammatory processes. We hypothesized that ELF3 modulates MARK4 expression in vascular endothelial cells, thus contributing to high glucose-mediated NLRP3 inflammasome activation. Plasma IL-1β, IL-18, NLRP3 inflammasome and MARK4 expression was increased in diabetic patients and rats. An in vitro study indicated that high glucose increased IL-1β and IL-18 expression and activated the NLRP3 inflammasome via upregulation of MARK4 in human umbilical vein endothelial cells (HUVECs). Furthermore, high glucose increased ELF3 expression. ELF3 downregulation reversed the effects of high glucose treatment. Accordingly, the effects of ELF3 overexpression were similar to those of high glucose treatment and were counteracted by siMARK4. Furthermore, ELF3 was found to interact with SET8. High glucose inhibited SET8 expression and histone H4 lysine 20 methylation (H4K20me1), a downstream target of SET8. Overexpression of SET8 inhibited high glucose-induced MARK4 expression and NLRP3 inflammasome activation. The effects of shSET8 were similar to those of high glucose treatment and were counteracted by siMARK4. A mechanistic study found that ELF3 and H4K20me1 were enriched in the MARK4 promoter region. si-ELF3 attenuated MARK4 promoter activity and augmented the inhibitory effect of SET8 on MARK4 promoter activity. Furthermore, SET8 downregulation and ELF3 upregulation were confirmed in diabetic patients and rats. In conclusion, ELF3 interacted with SET8 to modulate MARK4 expression, which participated in hyperglycaemia-mediated endothelial NLRP3 inflammasome activation.

## Introduction

Macrovascular and microvascular disorders have been recognized as important complications in diabetic patients^1^. Vascular endothelial injury is a direct result of hyperglycaemia and an important link to vascular disorders in diabetic patients^2^. High glucose-mediated endothelial injury has been indicated to include endothelial inflammation^3^.

Activation of the NOD-like receptor pyrin domain 3 (NLRP3) inflammasome leads to the generation of bioactive interleukin (IL)-1β and IL-18^4,5^, thus participating in endothelial inflammation and cardiovascular complications^6^. The NLRP3 inflammasome consists of three parts: NLRP3, caspase-1, and apoptosis-associated speck-like protein (ASC). Microtubule affinity regulating kinase 4 (MARK4) has been reported to play a crucial role in the regulation of NLRP3 inflammasome activation^7,8^ and may be a potential target for hyperglycaemia-mediated NLRP3 inflammasome activation and endothelial inflammation.

E74-like ETS transcription factor 3 (ELF3), also known as ESE-1, was originally identified as an epithelial-restricted ETS factor^9^. In response to inflammatory stimuli, ELF3 expression has been found to be increased in vascular endothelial cells^10^. Moreover, ELF3 has been reported to participate in endothelial inflammatory processes^10,11^. In the present study, we hypothesized that ELF3 may increase MARK4 expression, thus playing a crucial role in hyperglycaemia-induced NLRP3 inflammasome activation in vascular endothelial cells. More importantly, we explored the potential mechanism by which ELF3 modulates MARK4 expression.

## Material and Methods

### Subjects

Fifty newly diagnosed type 2 diabetes mellitus (T2DM) patients with poor glycaemic control and thirty generally healthy controls were consecutively recruited. The study complied with the Declaration of Helsinki and was approved by the Ethics Committee of Huzhou First People’s Hospital (license number: 20191209-01), and written informed consent was obtained from all subjects. The definition of T2DM was as follows: fasting plasma glucose levels ≥7 mmol/L, HbA1c levels ≥6.5%, plasma glucose levels after 2 hr ≥ 11.1 mmol/L or a random plasma glucose level ≥11.1 mmol/L. The exclusion criteria consisted of advanced liver disease, renal failure, cancer, valvular heart disease, severe heart failure, stroke, atrial fibrillation, peripheral arterial disease and other vascular diseases.

### Venous blood samples

Fasting venous blood samples from all subjects were collected in EDTA vacutainer tubes. The blood samples were centrifuged at 3000 rpm for 20 min at 4 °C, and then plasma samples were kept frozen at −80 °C until analysis.

### Isolation of peripheral blood mononuclear cells (PBMCs)

PBMCs were isolated by Ficoll standard density gradient centrifugation. The upper layer containing PBMCs was harvested and washed with Hank’s balanced salt solution and then with PBS. PBMCs were kept frozen at −80 °C until analysis.

### Animals

Four-week-old male Sprague Dawley rats weighing 150–200 g were used for the present experiments. The animals were obtained from Shanghai SLAC Laboratories and housed in a temperature-controlled environment (20–22 °C) under a 12 h light/dark cycle. Experiments were performed according to the Guide for the Care and Use of Laboratory Animals of Zhejiang University Laboratory Animal Welfare Ethics Review Committee and in accordance with the Institutional Guidelines for Animal Research and complied with the Guide for the Care and Use of Laboratory Animals published by the US NIH (2011). After a two-week adaptation period, 10 rats were randomly allocated into 2 groups as follows: Control group (con, *n* = 5), in which rats were injected intraperitoneally once with citrate buffer only (0.1 M, pH 4.5), and Diabetic group (DM, *n* = 5), in which rats received a high-fat diet for 2 weeks followed by a single intraperitoneal injection of streptozotocin (STZ, 50 mg/kg) and were moved back to a standard laboratory chow for 4 weeks. Hyperglycaemia was confirmed one week post STZ injection by measuring blood glucose through tail-neck blood sampling. When assessing experimental outcomes, the investigators were blinded to the treatments.

### Cell culture and reagents

Human umbilical vein endothelial cells (HUVECs) were obtained from American Type Culture Collection (ATCC; Manassas, USA) and cultured in Dulbecco’s modified Eagle medium (DMEM) with 5 mM glucose and supplemented with 10% foetal bovine serum at 37 °C in a humidified 5% carbon dioxide incubator.

For high glucose treatment, cells were washed with PBS twice to remove the complete medium and further cultured in DMEM with 25 mM glucose for 6 days with 10% foetal bovine serum at 37 °C in a humidified 5% carbon dioxide incubator. In addition, 5 mM glucose plus 20 mM mannitol (Sigma-Aldrich) was used as an osmotic control.

### Immunohistochemistry (IHC)

Standard IHC procedures were employed with anti-SET8 (ProteinTech, 14063-1-AP, 1/1000), anti-ELF3 (NOVUS, NBP1-30873, 1/1000) and anti-MARK4 (CST, 4834 S, 1/1000) antibodies. Rat aorta tissues were embedded in paraffin and processed for IHC. Sections were incubated with primary antibodies overnight at 4 °C in a humidified chamber. An EnVision^TM^ Detection Kit (Glostrup, Denmark) was used to detect signals according to the manufacturer’s instructions with diaminobenzidine (DAB) as the enzyme substrate.

### Co-immunoprecipitation (CoIP) and Immunocytochemistry/Immunofluorescence (ICC/IF)

Whole-cell protein lysates were extracted with a cell lysis buffer containing PMSF (Beyotime Biotechnology, Shanghai), and 30 μl of the lysates was removed as input. For endogenous IP, lysates were incubated with the corresponding primary antibodies and 50 μl protein A/G Dynabeads (ThermoFisher, USA) at 4 °C overnight. Then, 10 μl of input and IP fractions were subjected to western blotting. Cells (3 × 10^4^) were cultured in a laser confocal Petri dish (J40201, φ 20 mm, JingAn Biological, Shanghai) with DMEM containing 5 mM glucose or 25 mM glucose and 10% foetal bovine serum for 6 days at 37 °C in a humidified 5% carbon dioxide incubator. The cells were incubated in 100% methanol (chilled at −20 °C) at room temperature for 5 min. Then, 4% paraformaldehyde in PBS (pH 7.4) was added for 10 min at room temperature. Cells were washed three times with ice-cold PBS for 5 min each time. The samples were incubated for 10 min with PBS containing 0.3% Triton X-100. Cells were washed in PBS three times for 5 min. Cells were incubated with 3% BSA for 1 hour to block nonspecific binding of the antibodies. Cells were incubated with the diluted antibody (1:100) in 3% BSA overnight at 4°C. The solution was decanted, and the cells were washed three times in PBS for 5 min each wash. Cells were incubated with the secondary antibody in 3% BSA for 2 h at room temperature in the dark. The secondary antibody solution was decanted, and the cells were washed three times with PBS for 5 min each in the dark. Cells were incubated with 0.1–1 μg/ml DAPI for 1 min and rinsed with PBS. The coverslips were mounted with a drop of mounting medium. The coverslips were sealed with nail polish to prevent drying and movement under the microscope. The slides were stored in the dark at −20 °C or 4°C. The fixed samples were imaged by confocal microscopy. We mostly used an LSM 510 laser scanning confocal microscope for imaging (Carl Zeiss, Thornwood, NY) and a Plan-Apo 60×/1.4 NA oil lens objective. Images of cells in each channel and at multiple frames of view were acquired using a 1.4-megapixel cooled extended spectra range RGB digital camera set at 1024 × 1024 resolution (Carl Zeiss, Thornwood, NY).

### Chromatin immunoprecipitation (ChIP)

ChIP assays were implemented with a Simple ChIP Plus Sonication Chromatin IP Kit (Cell Signaling Technology, MA) according to the manufacturer’s instructions. In brief, HUVECs were fixed with 1% formaldehyde for 10 min at room temperature to mediate DNA and protein cross-linking. Glycine was then used to terminate the DNA and protein cross-linking reaction. Chromatin was sheared with the use of a Microson Ultrasonic Cell Disruptor XL (Misonix). Ten microliters of the sonicated solution was collected from each sample as input, and the remaining sample was incubated with anti-ELF3 (NOVUS, NBP1-30873), and anti-histone H4 lysine 20 methylation (H4K20me1; Abcam, ab9051) antibodies or a negative control IgG at 4 °C overnight. Immunoprecipitants were bound to protein G magnetic beads, and the DNA-protein cross link was reversed by incubating at 65°C for 2 h. Then, the DNA was purified, and enriched DNA sequences were analysed by qPCR. MARK4 oligonucleotide sequences for PCR primers were as follows: forward 5′-CCAACTGGGGAGAGAATGGG-3′ and reverse 5′-AGACTGAGAGAGACCCCAC-3′.

### Dual-luciferase assay

A Promega Dual-Luciferase Assay Kit (Madison, WI, United States) was used to assess the impact of SET8 and ELF3 on MARK4 promoter activity. In brief, the promoter regions 2000 bp upstream from the transcript start site (TSS) of the MARK4 gene were amplified from genomic DNA of HUVECs and ligated into a pGL3-Basic vector to generate the pGL3-MARK4 construct. pGL3-MARK4 was then co-transfected with a Renilla luciferase vector into HUVECs, and MARK4 promoter activity was determined by a dual-luciferase assay kit.

### Statistical analysis

For the present study, the sample sizes of animals and HUVECs were determined by an assessment of the magnitude of high glucose-induced MARK4 protein expression which was observed in our pilot experiments, and we anticipated that statistical significance could be achieved with the sample size of 5 in every experiment.

The results are presented as the mean ± SD (standard deviation) from at least five separately performed experiments. Two-tailed unpaired *t* tests or two-way ANOVA with GraphPad Prism Version 7.0 (GraphPad Software, San Diego, CA) were performed to compare the groups followed with Bonferroni-corrected pairwise comparisons. *P* < 0.05 was considered significant.

## Results

### NLRP3 inflammasome activation and MARK4 increase in diabetic patients and rats

**Fig. 1:**
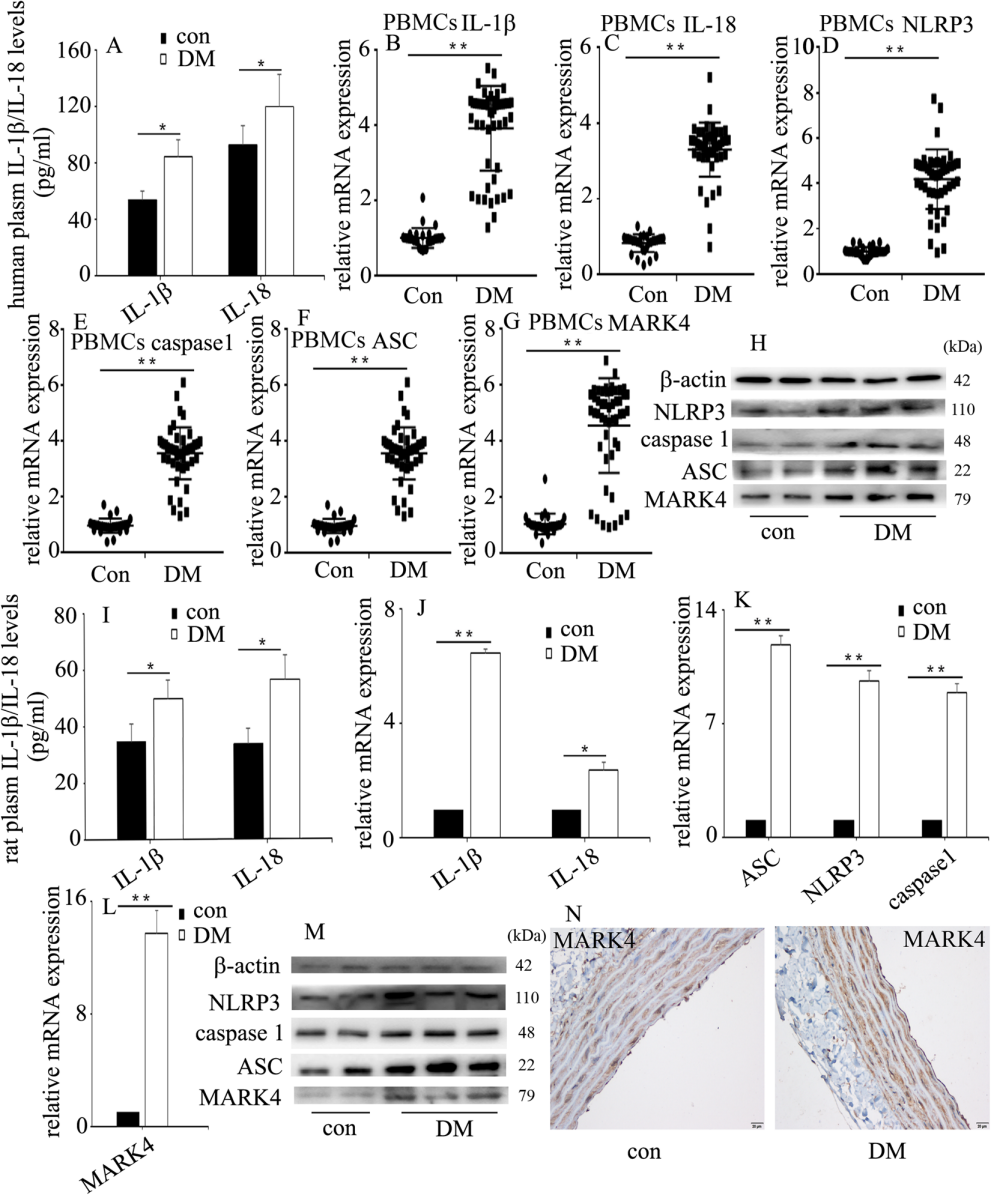
NLRP3 inflammasome activation and MARK4 increase in diabetic patients and rats. **a** Plasma interleukin (IL)-1β and IL-18 were measured in diabetic patients and heathy controls (con: *n* = 30, DM: *n* = 50). **b**–**g** The mRNA expression of IL-1β, IL-18, NLRP3, caspase 1, ASC and MARK4 was examined by qPCR in PBMCs from subjects (con: *n* = 30, DM: *n* = 50). **h** Results from western blot analysis of NLRP3, caspase 1, ASC and MARK4 expression in PBMCs from subjects (con: *n* = 30, DM: *n* = 50). **i** Plasma IL-1β and IL-18 were measured in the control group and diabetic group in rats (*n* = 5/group). **j**–**l** The mRNA expression of IL-1β, IL-18, NLRP3, caspase 1, ASC and MARK4 was examined by qPCR in aorta tissues from the control group and diabetic group in rats (*n* = 5/group). **m** Results from western blot analysis of NLRP3, caspase 1, ASC and MARK4 expression in aorta tissues from the control group and diabetic group in rats (*n* = 5/group). **n** Immunostaining of MARK4 in aorta tissues from the control group and diabetic group (*n* = 5/group). Scale bar, 20 μm. (*P ≤ 0.001, **P ≤ 0.0001, compared with the control group).

Endothelial NLRP3 inflammasome activation mediates endothelial inflammation^4,5^, thus resulting in vascular endothelial injury in hyperglycaemia^6^. The characteristics of the subjects are shown in Table 1. Fasting blood sugar and glycated haemoglobin (HbA1c) levels in patients newly diagnosed with T2DM were higher than those in healthy controls. In the present study, we found that plasma IL-1β and IL-18 were increased in diabetic patients (Fig. 1a). Previous studies have shown that MARK4 participates in NLRP3 inflammasome activation, thus mediating IL-1β and IL-18 expression^7,8^, so we detected NLRP3 inflammasome and MARK4 expression in diabetic patients. One previous study determined NLRP3 inflammasome expression in PBMCs to reflect the protein levels in the blood vessels of diabetic patients^12^. Therefore, in the present study, we detected IL-1β, IL-18, NLRP3 inflammasome and MARK4 expression in PBMCs in subjects. Compared with those in the healthy controls, the protein and/or mRNA levels of IL-1β, IL-18, NLRP3 inflammasome and MARK4 expression were increased in the PBMCs of diabetic patients (Fig. 1b–h).

**Table 1:** The baseline characteristics of the subjects.

Variables	Con	DM	*P* value
Male (%)	43	56	0.76
Age (years)	53.1 ± 12.9	58.3 ± 13.6	0.09
BMI (kg/m^2^)	23.2 ± 2.6	24.9 ± 4.2	0.41
SBP (mm Hg)	125.4 ± 13.3	126.7 ± 15.2	0.69
DBP (mm Hg)	69.7 ± 10.7	68.3 ± 9.7	0.53
FBS (mmol/L)	5.0 ± 0.6	14.1 ± 5.7	<0.0001
HbA1C (%)	5.6 ± 0.8	10.4 ± 2.5	<0.0001

*BMI* body mass index, *SBP* systolic blood pressure, *DBP* diastolic bllod pressure, *FBS* fasting blood sugar, *HbA1c* glycated hemoglobin

The blood glucose concentrations in diabetic rats were significantly higher than those in the control group (Supplementary Fig. 1). Similarly, plasma levels of IL-1β and IL-18 in diabetic rats (Fig. 1i), as well as protein and/or mRNA levels of IL-1β, IL-18, NLRP3 inflammasome and MARK4 in aorta tissues of diabetic rats, were higher than those of the control group (Fig. 1j–n).

### High glucose mediated NLRP3 inflammasome activation and endothelial inflammation via upregulation of MARK4 expression in HUVECs

**Fig. 2:**
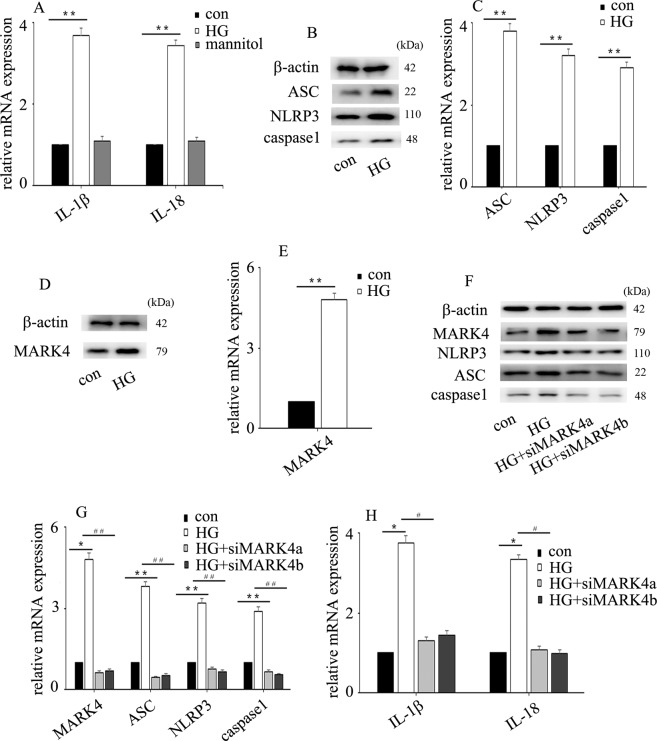
High glucose mediated endothelial NLRP3 inflammasome activation via upregulation of MARK4 expression in HUVECs. **a** Interleukin (IL)-1β and IL-18 mRNA expression in normal and hyperglycaemic HUVECs. **b** Western blot analysis of NLRP3 inflammasome expression in normal and hyperglycaemic HUVECs. **c** The mRNA expression of NLRP3 inflammasome in normal and hyperglycaemic HUVECs. **d** Western blot analysis of MARK4 expression in normal and hyperglycaemic HUVECs. **e** The mRNA expression of MARK4 in normal and hyperglycaemic HUVECs. **f** The effects of siMARK4 on high glucose-induced NLRP3 inflammasome protein expression in hyperglycaemic HUVECs. **g** The effects of siMARK4 on high glucose-induced NLRP3 inflammasome mRNA expression in hyperglycaemic HUVECs. **h** The effects of siMARK4 on high glucose-induced IL-1β and IL-18 mRNA expression in hyperglycaemic HUVECs. (*P ≤ 0.001, **P ≤ 0.0001, compared with the control group; ^#^*P* ≤ 0.001, ^##^*P* ≤ 0.0001, compared with the high glucose treatment group, *n* = 5/group).

To determine whether high glucose could induce NLRP3 inflammasome activation in HUVECs, cells were sub-incubated in different types of media, normal glucose (con, 5 mM, 6 days) and high glucose (HG, 25 mM, 6 days). The results indicated that high glucose increased IL-1β and IL-18 mRNA expression (Fig. 2a) and increased NLRP3, caspase 1 and ASC expression at the protein (Fig. 2b) and mRNA (Fig. 2c) levels in HUVECs. Mannitol had no effect on IL-1β or IL-18 expression (Fig. 2a). Previous studies have shown that MARK4 participates in NLRP3 inflammasome activation^7,8^, so we detected MARK4 expression in HUVECs. We found that high glucose treatment augmented MARK4 expression (Fig. 2d, e). To further confirm that MARK4 was involved in NLRP3 inflammasome activation in hyperglycaemic HUVECs, we used two independent siRNAs against MARK4. The effects of siMARK4 were confirmed by western blotting (Fig. 2f) and quantitative real-time PCR (Fig. 2g). The results showed that siMARK4 decreased high glucose-induced NLRP3 inflammasome activation (Fig. 2f, g) and inhibited high glucose-induced IL-1β and IL-18 mRNA expression in hyperglycaemic HUVECs (Fig. 2h). These data indicated that MARK4 positively regulated NLRP3 inflammasome activity, thus mediating endothelial IL-1β and IL-18 production in hyperglycaemic HUVECs.

### ELF3 upregulation participated in high glucose-induced endothelial NLRP3 inflammasome activation via increase of MARK4 expression in HUVECs

**Fig. 3:**
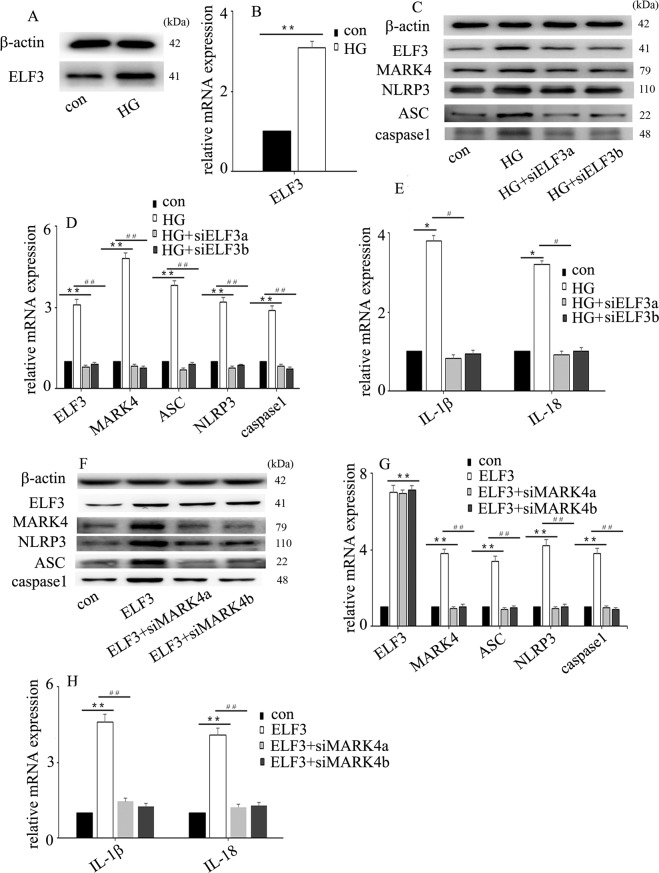
ELF3 upregulation participated in high glucose-induced endothelial NLRP3 inflammasome activation via increase of MARK4 expression in HUVECs. **a** Western blot analysis of ELF3 expression in normal and hyperglycaemic HUVECs. **b** The mRNA expression of ELF3 in normal and hyperglycaemic HUVECs. **c** The effects of siELF3 on high glucose-induced MARK4 and NLRP3 inflammasome protein expression in hyperglycaemic HUVECs. **d** The effects of siELF3 on high glucose-induced MARK4 and NLRP3 inflammasome mRNA expression in hyperglycaemic HUVECs. (**e**) The effects of siELF3 on high glucose-induced interleukin (IL)-1β and IL-18 mRNA expression in hyperglycaemic HUVECs. **f** The effects of siMARK4 on ELF3 overexpression-induced NLRP3 inflammasome protein expression in HUVECs. **g** The effects of siMARK4 on ELF3 overexpression-induced NLRP3 inflammasome mRNA expression in HUVECs. **h** The effects of siMARK4 on ELF3 overexpression-induced IL-1β and IL-18 mRNA expression in HUVECs. (*P ≤ 0.001, **P ≤ 0.0001, compared with the control group; ^#^*P* ≤ 0.001, ^##^*P* ≤ 0.0001, compared with the high glucose treatment group, *n* = 5/group).

Previous studies have indicated that ELF3 participates in endothelial inflammatory processes^10,11^. Accordingly, ELF3 was found to be upregulated by high glucose treatment in this study (Fig. 3a, b). To investigate the effect of ELF3 on high glucose-induced NLRP3 inflammasome activation, both loss- and gain-of-function approaches were employed. Our data indicated that ELF3 downregulation counteracted the high glucose-induced MARK4 expression and endothelial inflammasome activation (Fig. 3c, d) and reversed the high glucose-induced IL-1β and IL-18 expression in HUVECs (Fig. 3e). Moreover, the effects of ELF3 overexpression were similar to those of high glucose treatment (Fig. 3f–h). To explore whether the effects of overexpression of ELF3 were achieved via upregulation of MARK4 expression, we knocked down MARK4 in ELF3-overexpressing HUVECs. The results showed that MARK4 downregulation reversed ELF3 overexpression-mediated endothelial NLRP3 inflammasome activation (Fig. 3f, g) and counteracted ELF3 overexpression-induced IL-1β and IL-18 expression in HUVECs (Fig. 3h). These data indicated that ELF3 overexpression mediated endothelial NLRP3 inflammasome activation via upregulation of MARK4 expression in hyperglycaemic HUVECs.

### ELF3 interacted with SET8

**Fig. 4:**
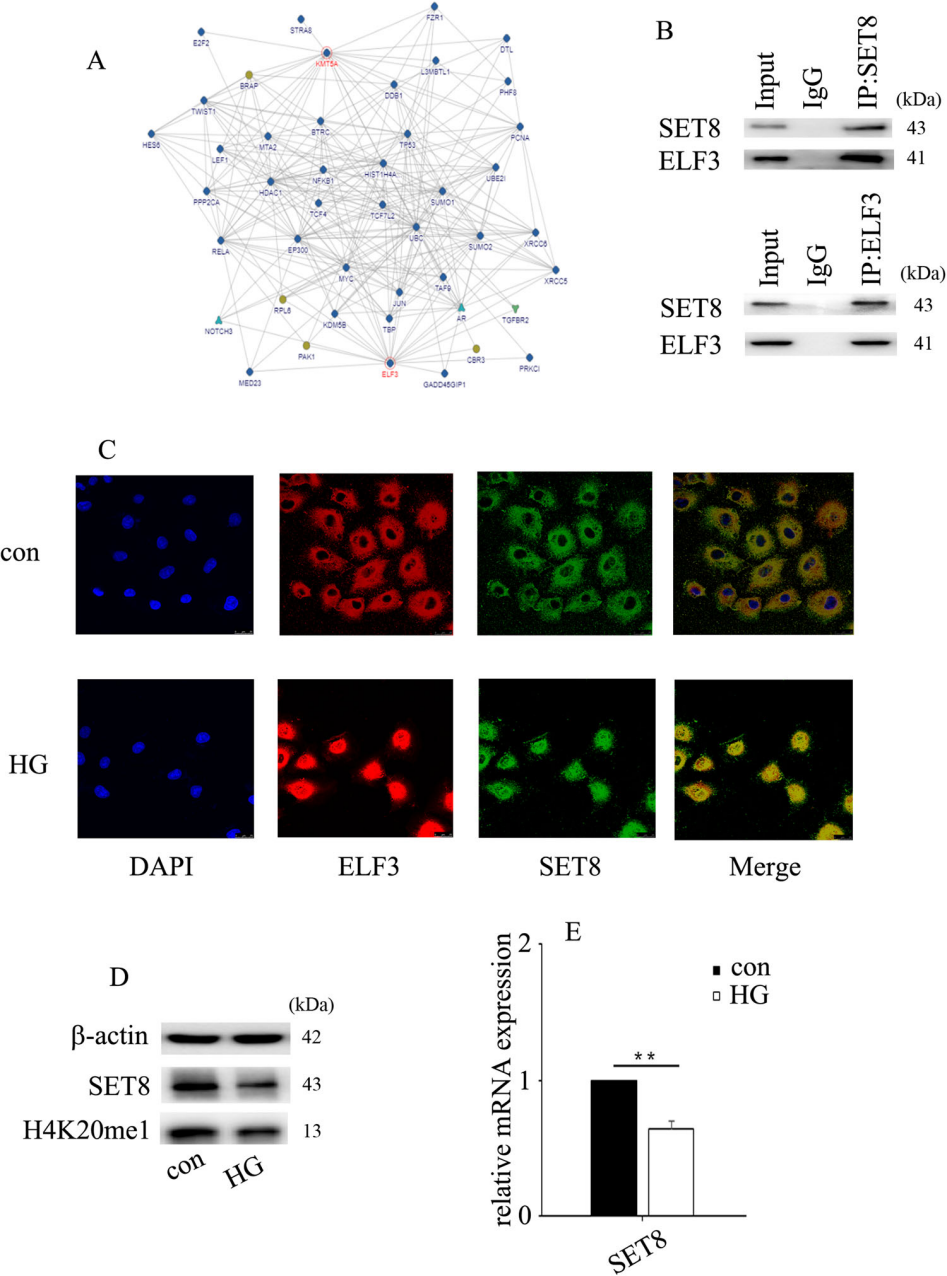
ELF3 interacted with SET8. **a** Several proteins that interact with ELF3 (https://string-db.org). **b** Interaction between ELF3 and SET8 in HUVECs was verified by co-IP. **c** Colocalization of ELF3 and SET8 in HUVECs by confocal microscopy. **d** Western blot analysis of SET8 expression in normal and hyperglycaemic HUVECs. **e** The mRNA expression of SET8 in normal and hyperglycaemic HUVECs. (*P ≤ 0.001, **P ≤ 0.0001, compared with the control group, *n* = 5/group).

To discover the potential regulatory mechanism, we predicted the proteins that interacted with ELF3 using bioinformatics. Several proteins that interact with ELF3 are shown in Fig. 4a (https://www.intomics.com/inbio/map). Our previous study indicated that SET8, also known as KMT5A, participates in high glucose-mediated endothelial inflammation in HUVECs^13^. Co-IP experiments verified the interaction between ELF3 and SET8 in HUVECs (Fig. 4b). Double immunofluorescent staining revealed co-localization of ELF3 and SET8 in plasmids and nuclei in vitro (Fig. 4c). Moreover, our data indicated that high glucose treatment induced SET8 and ELF3 nuclear translocation in HUVECs (Fig. 4c). Furthermore, we found that high glucose downregulated SET8 protein (Fig. 4d) and mRNA (Fig. 4e) expression in HUVECs. H4K20me1, a downstream target of SET8, was also decreased by high glucose treatment (Fig. 4d).

### SET8 downregulation participated in high glucose-induced endothelial NLRP3 inflammasome activation via upregulation of MARK4 expression in HUVECs

**Fig. 5:**
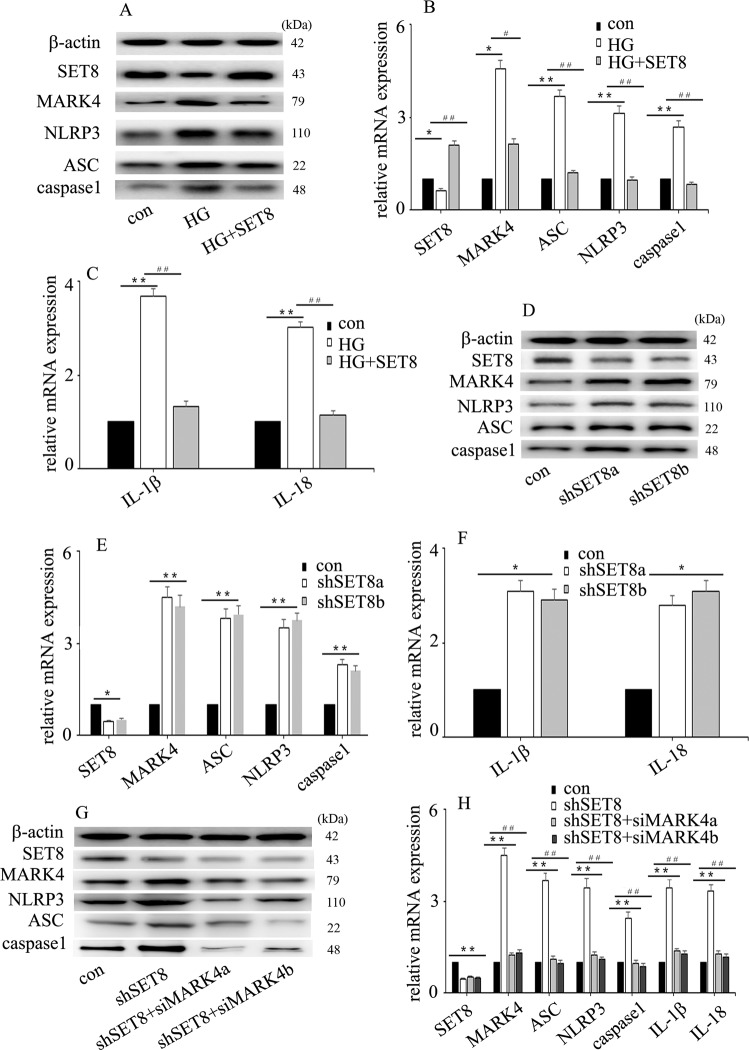
SET8 downregulation was involved in high glucose-induced endothelial NLRP3 inflammasome activation via upregulation of MARK4 expression in HUVECs. **a** The effects of SET8 overexpression on high glucose-induced MARK4 and NLRP3 inflammasome protein expression in hyperglycaemic HUVECs. **b** The effects of SET8 overexpression on high glucose-induced MARK4 and NLRP3 inflammasome mRNA expression in hyperglycaemic HUVECs. **c** The effects of SET8 overexpression on high glucose-induced interleukin (IL)-1β and IL-18 mRNA expression in hyperglycaemic HUVECs. **d** The effects of shSET8 on MARK4 and NLRP3 inflammasome protein expression in HUVECs. **e** The effects of shSET8 on MARK4 and NLRP3 inflammasome mRNA expression in HUVECs. **f** The effects of shSET8 on IL-1β and IL-18 mRNA expression in HUVECs. (*P ≤ 0.001, **P ≤ 0.0001, compared with the control group; ^#^*P* ≤ 0.001, ^##^*P* ≤ 0.0001, compared with the high glucose treatment group) **g** The effects of siMARK4 on shSET8-induced MARK4 and NLRP3 inflammasome protein expression in HUVECs. **h** The effects of siMARK4 on shSET8-induced MARK4, NLRP3 inflammasome, IL-1β and IL-18 mRNA expression in HUVECs. (*P ≤ 0.001, **P ≤ 0.0001, compared with the control group; ^#^*P* ≤ 0.001, ^##^*P* ≤ 0.0001, compared with the shSET8 treatment group, *n* = 5/group).

To investigate the effect of SET8 on high glucose-mediated endothelial NLRP3 inflammasome activation in HUVECs, both loss- and gain-of-function approaches were employed. Our data indicated that SET8 overexpression counteracted high glucose-induced MARK4 expression and NLRP3 inflammasome activation (Fig. 5a, b). Moreover, SET8 overexpression reversed high glucose-induced IL-1β and IL-18 expression in HUVECs (Fig. 5c). Furthermore, the effects of shSET8 were similar to those of high glucose treatment (Fig. 5d–f). To explore whether the effects of shSET8 were achieved via upregulation of MARK4 expression, we knocked down MARK4 in SET8-downregulated HUVECs. The results showed that MARK4 downregulation reversed SET8 silencing-induced NLRP3 inflammasome activation and counteracted shSET8-induced IL-1β and IL-18 expression (Fig. 5g, h). These data indicated that SET8 downregulation mediated endothelial inflammasome activation via upregulation of MARK4 expression.

### ELF3 interacts with SET8 to modulate MARK4 transcriptional activity in HUVECs

**Fig. 6:**
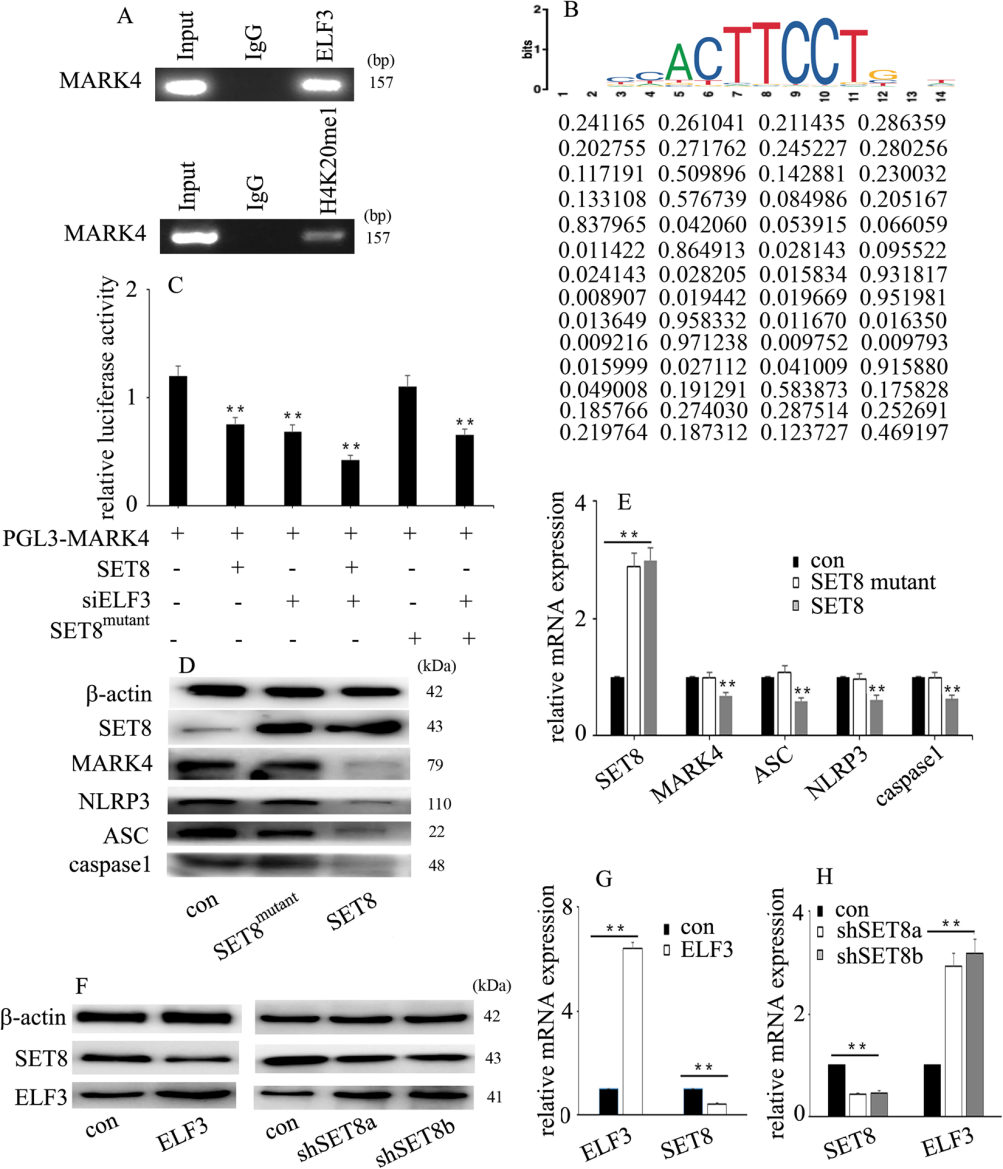
ELF3 interacted with SET8 to modulate MARK4 transcriptional activity in HUVECs. **a** ELF3 and H4K20me1 were enriched at the MARK4 promoter region. **b** The putative ELF3 binding site of MARK4. The motif logo and position weight matrix are shown in the upper and lower panels, respectively. **c** MARK4 promoter activity was determined by luciferase reporter assays. (*P ≤ 0.001, **P ≤ 0.0001, compared with the control group, *n* = 5/group). **d** Western blot analysis of SET8, MARK4 and NLRP3 inflammasome expression in control HUVECs, HUVECs overexpressing SET8 and HUVECs overexpressing mutant SET8^R259G^. **e** qPCR analysis of SET8, MARK4 and NLRP3 inflammasome expression in control HUVECs, HUVECs overexpressing SET8 and HUVECs overexpressing mutant SET8^R259G^ (*P ≤ 0.001, **P ≤ 0.0001, compared with the control group, *n* = 5/group). **f** The effects of ELF3 overexpression or SET8 downregulation on SET8 or ELF3 protein expression in HUVECs. **g** The effects of ELF3 overexpression on SET8 mRNA expression in HUVECs (*P ≤ 0.001, **P ≤ 0.0001, compared with the control group, *n* = 5/group). **h** The effects of SET8 downregulation on ELF3 mRNA expression in HUVECs. (*P ≤ 0.001, **P ≤ 0.0001, compared with the control group, *n* = 5/group).

Furthermore, to determine whether MARK4 is targeted by ELF3 and SET8, we examined the genome-wide distribution of ELF3 and H4K20me1 by ChIP assay in HUVECs. Our data indicated that both ELF3 and H4K20me1 were enriched in the MARK4 promoter region (Fig. 6a). The putative ELF3 binding site is shown in Fig. 6b. The motif logo and position weight matrix are shown in the upper and lower panels, respectively (Fig. 6b). The predicted binding region and the primer positions are shown in Supplementary Fig. 2. Moreover, luciferase reporter assays indicated that SET8 overexpression reduced MARK4 promoter activity but also increased the inhibitory effect of siELF3 on MARK4 promoter activity (Fig. 6c). The SET8^R259G^ mutant had no effect on MARK4 promoter activity (Fig. 6c). Furthermore, SET8 overexpression attenuated MARK4 and NLRP3 inflammasome expression, while mutant SET8^R259G^ did not affect MARK4 and NLRP3 inflammasome expression (Fig. 6d, e). SET8 is the only known lysine methyltransferase responsible for the specific monomethylation on H4K20me1^14^. These data demonstrate that ELF3 interacts with SET8 to modulate MARK4 promoter activity in HUVECs and that SET8-mediated H4K20me1 is necessary to regulate MARK4 expression in HUVECs. Furthermore, ELF3 overexpression inhibited SET8 expression (Fig. 6f, g), while SET8 downregulation increased ELF3 expression in HUVECs (Fig. 6f, h).

### SET8 decrease and ELF3 increase was confirmed in diabetic patients and rats

**Fig. 7:**
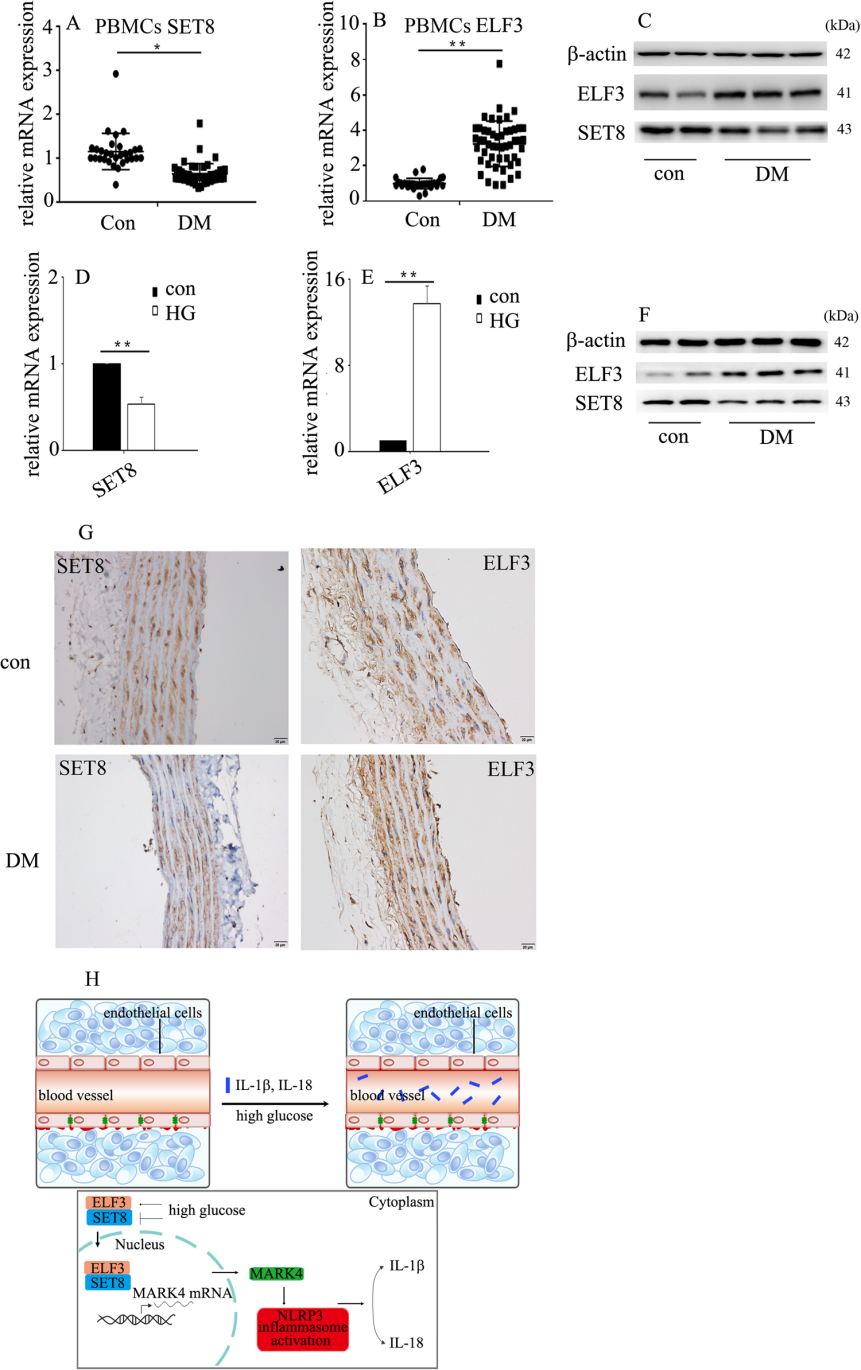
SET8 decrease and ELF3 increase were confirmed in diabetic patients and rats. **a** The mRNA expression of SET8 was examined by qPCR in PBMCs from diabetic patients and healthy controls (con: *n* = 30, DM: *n* = 50). **b** The mRNA expression of ELF3 was examined by qPCR in PBMCs from diabetic patients and healthy controls (con: *n* = 30, DM: *n* = 50). **c** Results from western blot analysis of ELF3 and SET8 expression in PBMCs from diabetic patients and heathy controls (con: *n* = 30, DM: *n* = 50). **d** The mRNA expression of SET8 was examined by qPCR in aorta tissues from the control group and diabetic group in rats (*n* = 5/group). **e** The mRNA expression of ELF3 was examined by qPCR in aorta tissues from the control group and diabetic group in rats (*n* = 5/group). **f** Results from western blot analysis of ELF3 and SET8 expression in aorta tissues from the control group and diabetic group in rats (*n* = 5/group). **g** Immunostaining of SET8 and ELF3 in aorta tissues from the control group and diabetic group (*n* = 5/group). Scale bar, 50 μm. **h** Schematic representation of the working model. High glucose mediated NLRP3 inflammasome activation via upregulation of MARK4 expression in vascular endothelial cells. Moreover, high glucose increased ELF3 expression while inhibiting SET8 expression. Furthermore, ELF3 interacted with SET8 to modulate MARK4 expression, which was involved in the high glucose-mediated endothelial inflammasome activation in hyperglycaemic HUVECs. (*P ≤ 0.001, **P ≤ 0.0001, compared with the control group).

To determine whether the protein and mRNA levels of SET8 and ELF3 in diabetic patients and rats were consistent with those of hyperglycaemic HUVECs, we detected SET8 and ELF3 expression in the PBMCs of diabetic patients and aorta tissues of diabetic rats. Our data indicate that SET8 decreased and ELF3 increased in the PBMCs of diabetic patients (Fig. 7a–c) and aorta tissues of diabetic rats (Fig. 7d–g). In conclusion, the present study indicated that ELF3 and SET8 interacted to modulate MARK4 expression, thus mediating endothelial NLRP3 inflammasome activation in hyperglycaemic vascular endothelial cells (Fig. 7h).

## Discussion

The main finding of the present study is that high glucose, via upregulation of MARK4 expression, induced NLRP3 inflammasome activation, thus mediating IL-1β and IL-18 expression in vascular endothelial cells. Moreover, the high glucose-mediated increase in ELF3 expression and reduction in SET8 expression were involved in MARK4 overexpression. Furthermore, H4K20me1 and ELF3 were enriched in the MARK4 promoter region. Mechanistic studies demonstrated that ELF3 interacted with SET8 to modulate MARK4 transcriptional activity in hyperglycaemic vascular endothelial cells.

Hyperglycaemia plays a crucial role in diabetes-mediated vascular complications. High glucose exposure leads to endothelial dysfunction, which is an important event in vascular complications. Hyperglycaemia-mediated endothelial dysregulation has been recognized as a potential target for the treatment of cardiovascular disorders in patients with diabetes^15^. NLRP3 inflammasome activation has been shown to be involved in the pathogenesis of cardiovascular disease and vascular endothelial dysfunction^6^. IL-1β and IL-18, two key cytokines regulated by NLRP3 inflammasome activation, participate in endothelial inflammation and cardiovascular disease in humans^7,16–19^. Inhibition of NLRP3 inflammasome activation inhibited high glucose-mediated IL-1β and IL-18 expression, thus attenuating high glucose-mediated endothelial inflammation^20^. Previous studies indicated that NLRP3 inflammasome activation was regulated by MARK4 expression^7,8^. In the present study, we found that high glucose increased MARK4 expression (Fig. 2d, e) and induced endothelial NLRP3 inflammasome activity (Fig. 2b, c). Moreover, siMARK4 reversed high glucose-mediated endothelial inflammasome activation in HUVECs (Fig. 2f, g). These data demonstrated that high glucose mediated the endothelial NLRP3 inflammasome activation via upregulation of MARK4 expression.

ELF3 was originally identified as an epithelial-restricted ETS factor^9^. In response to inflammatory stimuli, ELF3 expression is increased in vascular endothelial cells^10^. ELF3 can activate cyclooxygenase 2^11^, inducible nitric oxide synthase^10^ and angiopoietin-1^21^ gene transcription, thus participating in endothelial inflammatory processes. In addition to mediating vascular endothelial inflammation, ELF3 also participated in vascular remodelling in angiotensin II-treated mice^22^. As ELF3 is an inducer of endothelial inflammation^10,11,21^, ELF3 inhibition may become a crucial strategy for the treatment of hyperglycaemia-mediated endothelial inflammation and injury. In the present study, ELF3 was upregulated upon high glucose treatment (Fig. 3a, b) and enriched at the MARK4 promoter region (Fig. 6a). Moreover, siELF3 reversed high glucose-induced MARK4 expression and endothelial inflammasome activation (Fig. 3c–e). Furthermore, siMARK4 counteracted ELF3 overexpression-induced NLRP3 inflammasome activation in HUVECs (Fig. 3f–h). These data indicated that ELF3 was involved in high glucose-induced NLRP3 inflammasome activation via augmentation of MARK4 expression.

SET8 is a member of the SET domain-containing methyltransferase family^23,24^, which specifically participates in the monomethylation of lysine 20 of histone H4 (H4K20)^14^. The methyltransferase activity of SET8 is involved in a variety of crucial cellular signalling pathways, including DNA damage, cell cycle progression, transcriptional and post-translational regulation, and cellular metabolism^24,25^. Our previous study indicated that SET8 participated in the high glucose-mediated expression of endothelial adhesion molecules and proinflammatory enzymes, thus inducing endothelial inflammation in HUVECs^13,26^. In this study, SET8 overexpression attenuated high glucose-induced MARK4 expression and NLRP3 inflammasome activation (Fig. 5a, b), thus improving high glucose-induced IL-1β and IL-18 expression (Fig. 5c). Moreover, H4K20me1, a downstream target of SET8, was enriched at the MARK4 promoter region (Fig. 6a). siMARK4 counteracted SET8 knockdown-induced MARK4 expression (Fig. 5g, h). These data indicated that SET8 overexpression inhibited high glucose-induced NLRP3 inflammasome activation via inhibition of MARK4.

The present study demonstrated that ELF3 interacts with SET8 and occupies the MARK4 promoter region. Luciferase reporter assays indicated that SET8 overexpression reduced MARK4 promoter activity but also increased the inhibitory effect of siELF3 on MARK4 promoter activity (Fig. 6c). To further confirm the results, we constructed a mutant SET8^R259G^ plasmid that had a dominant-negative role in H4K20me1^26^. Our data showed that mutant SET8^R259G^ did not affect MARK4 or NLRP3 inflammasome expression. These data indicated that ELF3 interacted with SET8 to regulate MARK4 expression. Moreover, SET8-mediated H4K20me1 participated in the modulation of MARK4 transcription.

The present study has some limitations. First, the mechanistic study was mainly carried out in HUVECs and should be further confirmed in studies in vivo. Second, the mechanism by which ELF3 interacts with SET8 needs further clarification. Third, this study was performed in HUVECs and should be validated in other primary endothelial cell models. Fourth, the mechanism by which SET8 and ELF3 inhibit each other deserves further research.

In summary, the present study demonstrated that high glucose induced NLRP3 inflammasome activation via upregulation of MARK4 expression in vascular endothelial cells. Moreover, high glucose increased ELF3 expression while inhibiting SET8 expression. Furthermore, ELF3 interacted with SET8 to modulate MARK4 expression, which was involved in high glucose-mediated endothelial inflammasome activation in hyperglycaemic HUVECs.

## Supplementary information

SUPPLEMENTAL-methods

SUPPLEMENTAL-figure 1

SUPPLEMENTAL-figure 2

SUPPLEMENTAL-Table 1

SUPPLEMENTAL-figure legend
