# Supplementary material for: High glucose mediates NLRP3 inflammasome activation via upregulation of ELF3 expression
Source: Cell Death Dis. 2020 May 21;11(5):383. doi: 10.1038/s41419-020-2598-6 (PMC7242464; doi:10.1038/s41419-020-2598-6)
Supplement: Supplementary file 1 — SUPPLEMENTAL-methods [file 41419_2020_2598_MOESM1_ESM.docx]

**Supplementary Material and Methods**

**Preparation of blood from rats for analysis**

At the end of the experimental period, blood samples were collected by cardiac puncture under anaesthesia (thiopental sodium at 40 mg/kg body weight), and the animals were then culled. The blood samples were centrifuged at 3000 rpm for 20 min at 4°C, and then plasma samples were kept frozen at −80°C until analysis.

**Detection of IL-1β and IL-18 levels**

 Human and rat plasma IL-1β and IL-18 were measured using enzyme-linked immunosorbent assay (ELISA) kits from Meimian Industrial Co., Ltd. (Jiangsu, China).

**Plasmid construction, transfection and siRNA treatments**

The Flag-tagged coding sequence of human SET8 was cloned into the lentiviral vector pPCDH-CMV-MCS-EF1-puro to generate the SET8 expression plasmid. The SET8 plasmid was transfected into HUVECs using Lipofectamine 3000 (Invitrogen, Waltham, USA) according to the manufacturer’s instructions. At 48 h after transfection, cells were harvested for western blot and qPCR analysis and then subjected to subsequent experiments. HUVECs were also transfected with shRNA against SET8, siRNA against ELF3 and siRNA against MARK4 using Lipofectamine 3000 (Invitrogen, USA) according to the manufacturer’s instructions.

The sequences of SET8 shRNA (Biotend, Shanghai) were as follows: shRNA-a, 5'-CAACAGAATCGCAAACTTA-3' and shRNA-b, 5'-CAACAGAATCGCAAACTTA-3'.

The sequences of ELF3 siRNAs (Biotend, Shanghai) were as follows:

siRNA#a, sense, 5'-GCCAUUGACUUCUCACGAUdTdT-3’, anti-sense, 5'-AUCGUGAGAAGUCAAUGGCdTdT-3' and

siRNA#b, sense, 5'-GCCAUGAGGUACUACUACAdTdT-3', anti-sense, 5'-UGUAGUAGUACCUCAUGGCdTdT-3'.

The sequences of MARK4 siRNA (Biotend, Shanghai) were as follows:

siRNA#a, sense, 5'-CCAUCAAGAUUAUCGACAAdTdT-3’, anti-sense, 5'-UUGUCGAUAAUCUUGAUGGdTdT-3' and

siRNA#b, sense, 5'-GCACUAUUGUCACCAGAAAdTdT-3', anti-sense, 5'-UUUCUGGUGACAAUAGUGCdTdT-3'.

**Western blot analysis**

Whole-cell extracts were prepared with Cell Lysis Buffer (Cell Signaling Technology, Danvers, USA). Protein samples were boiled in sample loading buffer for 10 minutes, and equal amounts of proteins from different groups of HUVECs were separated by 10% SDS-PAGE and transferred to PVDF membranes (Millipore, Billerica, USA). Membranes were blocked with 5% fat-free milk solution for 1 h, and then the membranes were incubated with primary antibodies overnight at 4°C. The primary antibodies used were monoclonal antibodies against β-actin (ProteinTech, 60004-1-Ig, 1/5000), SET8 (ProteinTech, 14063-1-AP, 1/1000), ELF3 (NOVUS, NBP1-30873, 1/1000), MARK4 (CST, 4834S, 1/1000), ASC (ProteinTech, 10500-1-AP), NLRP3 (ProteinTech, 19771-1-AP, 1/1000), and caspase1 (ProteinTech, 22915-1-AP, 1/1000). After washing the membranes with phosphate-buffered saline containing 1:1000 Tween-20, the corresponding HRP-conjugated secondary antibody (1:5000) was added at room temperature for 1 hour, and the membrane was further washed with PBST 5 times for 7 minutes each time. Finally, the signals were detected using a LAS-4000 mini CCD camera (GE Healthcare). The density of the protein bands was analysed by Scan-gel-it software. Protein expression was normalized to β-actin expression.

**Total RNA extraction**

Total RNA was extracted using TRIzol (Tiangen, Beijing, China). Chloroform was added, and the lysates were centrifuged at 12,000 × g for 15 min at 4°C. The supernatant was transferred to a new centrifuge tube. RNA was precipitated with 100% ethanol. The samples were rinsed with 75% DEPC-alcohol. Each RNA sample was diluted in 30 μL RNase-free water. The quantity and quality of RNA were assessed using a NanoDrop 2000c Spectrophotometer (Thermo Fisher, USA).

**cDNA preparation and quantitative real-time PCR (qRT-PCR)**

cDNA was synthesized by reverse transcription using a TaKaRa PrimeScript® RT Reagent Kit with gDNA eraser. Briefly, a mixture containing 1 μg RNA, 2.0 μL 5× gDNA eraser buffer, and RNase-free water up to 10 μL was incubated at 42°C for 2 min. Then, 4.0 μL of 5× PrimeScript® Buffer2, 1.0 μL PrimeScript®RT Enzyme Mix I, 1.0 μL RT Primer Mix, and RNase-free water up to 20 μL were added to the mixture. The RT reaction mixture had a final volume of 20 μL and was incubated at 42°C for 15 min and 85°C for 5 s. The expression of candidate genes and β-actin was determined by qRT-PCR. PCR was run using a QuantStudio 7 Flex Real-Time PCR System (Applied Biosystems) starting with denaturation at 95°C for 30 s followed by 40 cycles of 95°C for 5 s, 60°C for 30 s (annealing), a terminal extension step at 95°C for 10 s, and a final holding stage at 4°C. Melting curve controls were run to ensure primer specificity. All of the reactions were run in triplicate. The primer sequences used are listed in Supplementary Table 1.
