# Supplementary figures and images for: High glucose mediates NLRP3 inflammasome activation via upregulation of ELF3 expression
Source: Cell Death Dis. 2020 May 21;11(5):383. doi: 10.1038/s41419-020-2598-6 (PMC7242464; doi:10.1038/s41419-020-2598-6)

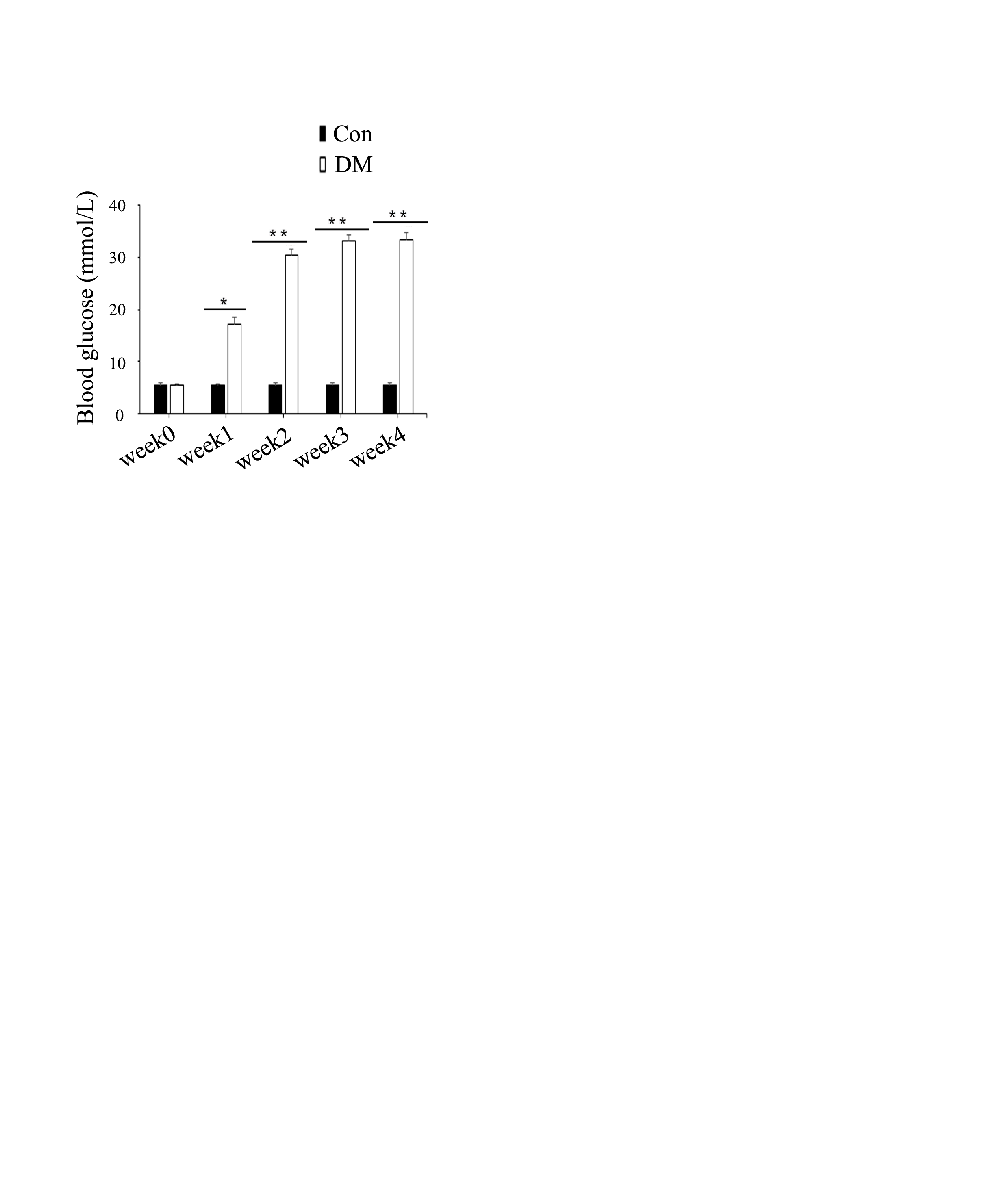

Supplement: Supplementary file 2 — SUPPLEMENTAL-figure 1 [file 41419_2020_2598_MOESM2_ESM.tif]

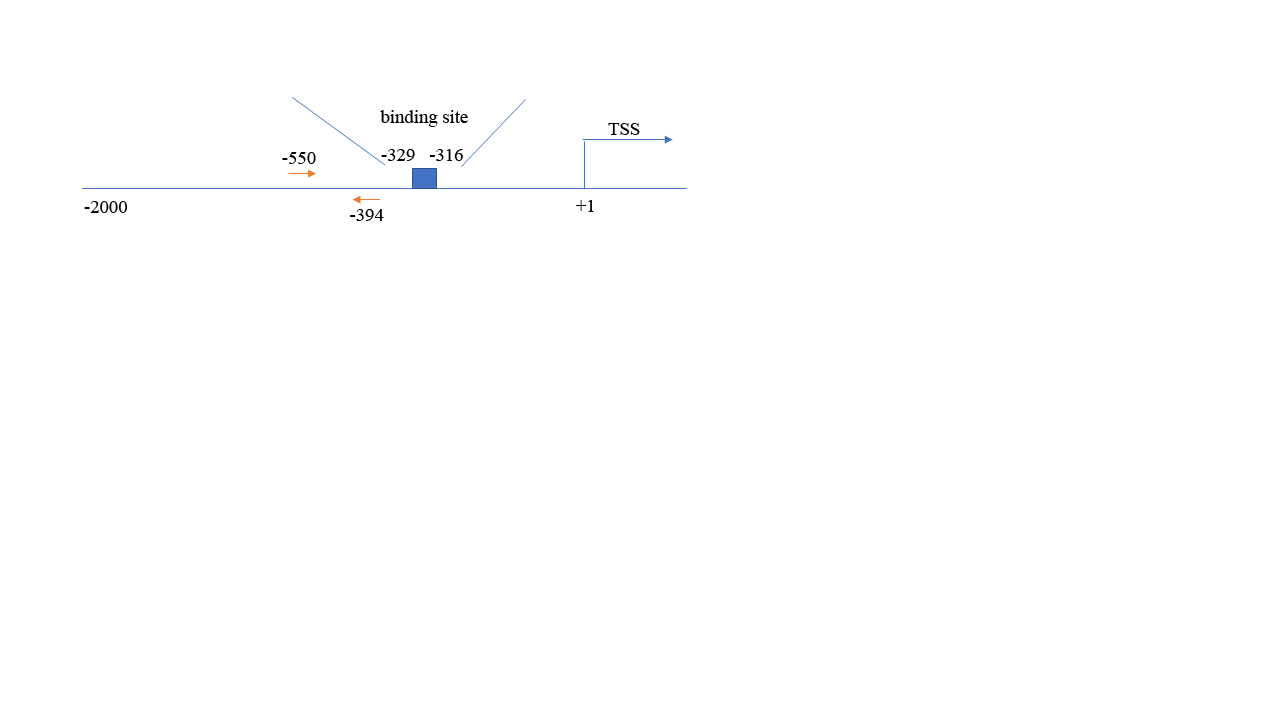

Supplement: Supplementary file 3 — SUPPLEMENTAL-figure 2 [file 41419_2020_2598_MOESM3_ESM.tif]
