# Supplementary material for: High glucose mediates NLRP3 inflammasome activation via upregulation of ELF3 expression
Source: Cell Death Dis. 2020 May 21;11(5):383. doi: 10.1038/s41419-020-2598-6 (PMC7242464; doi:10.1038/s41419-020-2598-6)
Supplement: Supplementary file 4 — SUPPLEMENTAL-Table 1 [file 41419_2020_2598_MOESM4_ESM.docx]

| species | RNA sequence |
| --- | --- |
| Human  β-actin  SET8  ELF3  ASC  MARK4  NLRP3  Caspase1  IL1  IL18  Rat  β-actin  SET8  ELF3  ASC  MARK4  NLRP3  Caspase1  IL1  IL18 | F 5’- CGGCTACAGCTTCACCACCAC -3’  R 5’- GCCATCTCTTGCTCGAAGTCCAG -3’  F 5’- TCCAGCAATCCTCCTCCTTCCTC -3’  R 5’- CCAGCCTAAGCAACAGATCCAGA -3’  F 5’- ATGGTTTTCGTGACTGCAAGAA -3’  R 5’- CAGTACTCTTTGCTCAGCTTTC -3’  F 5’- CTCAAGAAGTTCAAGCTGAAGC -3’  R 5’- TAGGTCTCCAGGTAGAAGCTG -3’  F 5’- GCCACCCACCCTCACTCTCC -3’  R 5’- CCCTGCTCCCATTCCCTCCTC -3’  F 5’- GCCCAAGGAGGAAGAGGAGGAG -3’  R 5’- TGGCGAGGAAGCAGGAGGAAG -3’  F 5’- GAAAAGCCATGGCCGACAAG -3’  R 5’- ATAGCTGGGTTGTCCTGCAC -3’  F 5’- GCCAGTGAAATGATGGCTTATT -3’  R 5’- AGGAGCACTTCATCTGTTTAGG -3’  F 5’- GCTGAAGATGATGAAAACCTGG -3’  R 5’- CAAATAGAGGCCGATTTCCTTG -3’  F 5’- CTTCCAGCCTTCCTTCCTGG -3’  R 5’- GAGCCACCAATCCACACAGA -3’  F 5’- GCAGGAAGAGAACTCCGTCG -3’  R 5’- AGAATCACATGACGGGGGTG -3’  F 5’- GCTGTACTCCACCTTGCAGA -3’  R 5’- AGTCTCCTAGGCCCTCTTGG -3’  F 5’- GCTCGTGGGTGACACATACA -3’  R 5’- AGCGTGTCATGGACTCTGTG -3’  F 5’- CTGTACACACTGGTCAGCGG -3’  R 5’- TTTCTTCCCGTGTGTAGCCC -3’  F 5’- AACTTGCAGAAGCTGGGGTT -3’  R 5’- CAGAACCTCACAGAGCGTCA -3’  F 5’- GAGGCAGACAGCTGGGTTAC -3’  R 5’- GAGTACGGCCATTGGTCTCC -3’  F 5’- ATAGCAGCTTTCGACAGTGAG -3’  R 5’- GCAGGTCGTCATCATCCCAC -3’  F 5’- GACCGAACAGCCAACGAATC -3’  R 5’- GTCCTGGCACACGTTTCTGA -3’ |

Supplementary Table 1 Primers used for real-time RT-PCR analysis.
