# Supplementary material for: High glucose mediates NLRP3 inflammasome activation via upregulation of ELF3 expression
Source: Cell Death Dis. 2020 May 21;11(5):383. doi: 10.1038/s41419-020-2598-6 (PMC7242464; doi:10.1038/s41419-020-2598-6)
Supplement: Supplementary file 5 — SUPPLEMENTAL-figure legend [file 41419_2020_2598_MOESM5_ESM.docx]

**Supplementary Data**

**Supplementary figure 1. Blood glucose levels in control and diabetic rats**

Blood glucose levels across the control and diabetic groups started after the induction of diabetes for 4 weeks. (*P ≤ 0.001, **P ≤ 0.0001, compared with the control group, n=5/group)

**Supplementary figure 2. The predicted binding region and the primer position**

The predicted binding region is from -329 bp to -316 bp, and the primer position is from -550 bp to -394 bp in the MARK4 promoter region.

**Supplementary Table 1.** Primers used for real-time RT-PCR analysis.
